# Cardiovascular risk assessment with SCORE2 and targeted health dialogues in primary care patients with mental illness

**DOI:** 10.1371/journal.pone.0348365

**Published:** 2026-05-04

**Authors:** Veronica Milos-Nymberg, Hanna Glock, Miriam Pikkemaat, Moa Wolff, Beata Borgström Bolmsjö, Peter Nymberg

**Affiliations:** 1 University Clinic Primary Care, Skåne University Hospital, Region Skåne, Sweden; 2 Center for Primary Health Care Research, Department of Clinical Sciences Malmö, Lund University, Malmö, Sweden; GSVM Medical College, INDIA

## Abstract

**Background:**

Patients with mental illness face a higher risk of cardiovascular disease (CVD), mainly due to unhealthy lifestyle habits. The CVD risk assessment tool Systematic COronary Risk Evaluation 2 (SCORE2) does not assess modifiable lifestyle factors beyond smoking. In Sweden, Targeted Health Dialogues (THD) are used in primary care as a structured, person-centred approach designed to evaluate lifestyle behaviours that influence CVD risk. Unlike SCORE2, the THD assesses a broader set of modifiable lifestyle-related cardiovascular risk factors, including BMI, waist–hip ratio, diet quality, physical activity, alcohol and nicotine use, sleep, and fasting biomarkers such as glucose and lipids. This study explored whether THD can add value in identifying cardiovascular risk factors in patients with mental illness, beyond what is captured by SCORE2.

**Method:**

This cross-sectional study included 120 patients, aged 40–69, with mental illness in Swedish primary care. CVD risk factors were assessed with a THD. SCORE2 was calculated, and the well-known CVD risk factors according to THD were compared across SCORE2 risk levels in men and women separately.

**Results:**

Most men (86.5%) and one-third (35.5%) of the women had moderate or high CVD risk according to SCORE2. Many patients with increased SCORE2 risk also had other risk factors identified through THD. A large proportion of both sexes with a low SCORE2 risk had several unhealthy modifiable risk factors (including high WHR, BMI, and physical inactivity).

**Conclusion:**

In conclusion, Targeted Health Dialogues may complement SCORE2 by identifying additional modifiable lifestyle-related risk factors relevant to cardiovascular prevention in patients with mental illness. Further research, including longitudinal and interventional designs, is warranted to evaluate whether THD-guided lifestyle counselling can reduce cardiovascular risk in this population.

## Introduction

The guidelines for cardiovascular prevention in Europe highlight the importance of using validated instruments in risk assessment [1]. At the beginning of the 21st century, the European Society of Cardiology developed the Systematic COronary Risk Evaluation (SCORE) instrument to predict the 10-year risk of developing fatal cardiovascular disease among European individuals without prior cardiovascular disease [2]. In 2021, SCORE2 was launched to update the original SCORE tool, providing a more accurate estimation of the 10-year risk for both fatal and non-fatal cardiovascular events [3].

However, neither SCORE nor SCORE2 considers lifestyle habits (except smoking) in the risk assessment, raising the question of whether important modifiable risk factors may be overlooked, and other risk assessment methods might be helpful.

Targeted Health Dialogue (THD) is a method developed to prevent non-communicable disease incidence and is offered to specific age groups in most regions of Sweden [4]. The THDs aim to identify lifestyle habits that are determinants for cardiovascular diseases (CVD) and type 2 diabetes mellitus (DM2), as well as their pre-stages. The Targeted Health Dialogue (THD) includes a comprehensive assessment of modifiable lifestyle‑related cardiovascular risk factors that are not captured by SCORE2, such as BMI, waist–hip ratio, diet quality, physical activity, alcohol consumption, nicotine use (smoking, snus, e‑cigarettes), sleep habits, and fasting biomarkers including glucose and lipid fractions. A recent Swedish review reported that according to GRADE (Grading of Recommendations, Assessment, Development and Evaluation), there was moderate evidence for the THD method in reducing premature all-cause and cardiovascular mortality as well as for reduction in blood pressure, cholesterol, fasting blood sugar, waist circumference, BMI, and improved dietary habits [5].

Individuals with mental illness have a higher risk for CVD, which is commonly attributed to a higher prevalence of hypertension, diabetes, hypercholesterolemia, and obesity [6–9]. This, in turn, may be due to unhealthy lifestyle behaviours [10–12]. Healthy lifestyle habits are known to reduce the risk of CVD [13–16], even in individuals with a heredity for CVD, which is a major risk factor [17]. In a pilot and feasibility study, using THDs among patients seeking care for mental illness in primary care, it was revealed that all the participants had at least one unhealthy habit, and many of them also had elevated levels of fasting blood sugar, lipids in blood, and blood pressure [10]. These findings are similar to a study comparing the risk of CVD between patients with bipolar disease (n = 65) and healthy controls (n = 29), highlighting the increased cardiovascular risk in this patient group regardless of mental illness severity [18]. The study showed impaired levels of lipids in blood, greater Body Mass Index (BMI) and Waist Hip ratio (WHR), a greater number of smokers, and an increased risk according to SCORE2 among those with bipolar disease [18]. Based on these previous findings, individuals with mental illness appear to require increased attention regarding early assessment and detection of cardiovascular risk factors. It is not clear yet if individuals seeking care for mental illness in a primary care setting are at high risk for cardiovascular disease, according to SCORE2. Furthermore, it is unclear how modifiable lifestyle behaviours are distributed among the SCORE2 risk categories in this population.

To our knowledge, this is the first study to evaluate SCORE2 in a primary care population seeking care for mental illness in Sweden and the first to directly compare SCORE2 with the Targeted Health Dialogue (THD) in this group.

### Aim

The main aim was to describe the SCORE2 risk level distribution in a population of patients seeking primary care due to mental illness.

A secondary aim was to report the distribution of modifiable lifestyle risk factors according to THD across the different risk levels of SCORE2.

### Method

In this cross-sectional study, 120 patients aged 40–69 years seeking care for mental illness (depression, anxiety, stress, or insomnia) at six publicly funded primary healthcare centres (PHCCs) in southern Sweden between February 24, 2020, and December 31, 2024, were opportunistically included. The participating PHCCs represented both urban and rural service areas with heterogeneous socioeconomic characteristics. During routine consultations for mental illness, any clinician (nurse, physician, or psychologist) could invite eligible patients, an approach intended to minimise recruitment bias and enhance the representativeness of real-world primary care. All included participants were invited to a THD.

#### Inclusion criteria.

- Age 40–69 years
- Seeking primary care for mental illness (depression, anxiety, stress, insomnia)
- Completed a Targeted Health Dialogue (THD)
- Availability of all necessary SCORE2 variables

#### Exclusion criteria.

- Severe mental illness requiring specialist psychiatric care
- Missing essential SCORE2 variables
- Cognitive impairment preventing informed consent

All lifestyle-related cardiovascular risk factors assessed in the THD, as well as all psychiatric diagnoses represented in the study population, are listed in S1 Table.

Before the THD, the participants measured BMI, WHR, and blood pressure. Blood sampling for analysis of fasting blood lipids and blood sugar was also collected. The participants completed an extensive survey about lifestyle habits, such as nutrient intake, physical activity, alcohol consumption, use of nicotine products, sleeping habits, dental status, and family history of DM2 and CVD. Based on a pedagogical visual colourful curve that describes the risk associated with specific lifestyle habits, a specially trained health dialogue leader (nurse, physiotherapist, dietitian or physician) discussed potential lifestyle improvements with the patient to reduce the risk of incident disease. Following the THD result, the patient was referred to another personnel category for further counselling and support if needed (e.g., dietary advice, motivation to increase physical activity, smoking cessation) [15,16,19,20].

In the THD, the healthy limits of WHR are considered as ≤.85 for women and ≤.90 for men. Participants with at least one first-degree relative affected by diabetes mellitus type 2 were classified as having a hereditary predisposition to the disease. In the THD, a family history of cardiovascular disease was considered significant if it occurred in male first-degree relatives before age 70 or in female first-degree relatives before age 75.

The healthy HDL limit in the THDs was set to ≥1.0 for men and ≥1.3 for women. High alcohol consumption was defined as more than 10 standard drinks per week or more than four drinks on a single occasion, with one standard drink equivalent to 4 millilitres of 40% alcohol. The scoring for dietary habits was calculated according to the Nordic Nutrition Recommendations 2023 [20].

We calculated the SCORE2 risk accordingly, using the participants’ values for non-HDL cholesterol, systolic blood pressure, smoking status, age, and sex. The different levels of risk according to SCORE2 were classified as low [1], moderate [2], and high [3] as presented in Table 1 [3].

**Table 1:** Categorisation of the 10-year risk of cardiovascular event in the different SCORE2 risk groups.

Risk	Low [1]	Moderate [2]	High [3]
<50 years	<2.5%	2.5 – < 7.5%	≥ 7.5%
50-69 years	<5%	5 – < 10%	≥ 10%

### Ethics approval and consent to participate

This study was conducted in accordance with the principles outlined in the Declaration of Helsinki and was approved by the Swedish Ethical Review Authority (2019–04990). Participants provided written informed consent prior to their inclusion in the study.

### Clinical trial registration

The HEAD-MIP study was registered at ClinicalTrials.gov on January 6th, 2022, with registration number NCT05181254.

### Statistics

As SCORE2 is validated for individuals aged 40–69 years, analyses were done for participants within this age range. All numeric variables were described by mean, median, standard deviation, and minimum and maximum values. We compared the percentage distribution across different levels of risk according to SCORE2 using the Kruskal-Wallis rank test as the data were not normally distributed. We used a cutoff for all variables based on international recommendations or guidelines for healthy levels, not the treatment cutoffs [21]. All analyses were done in STATA/SE 18.0.

## Results

**Fig 1:**
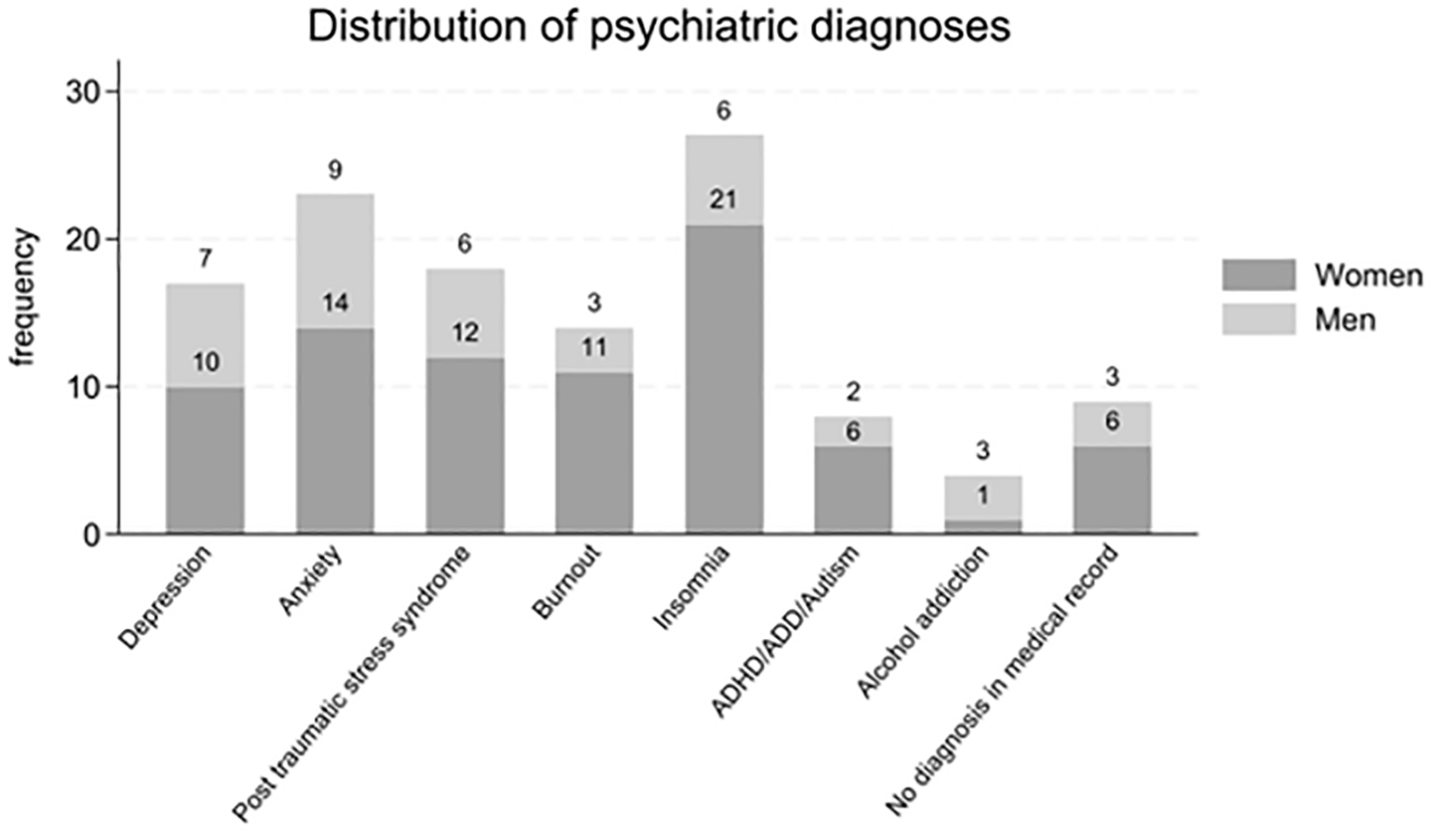
The distribution of psychiatric ICD-10 diagnoses documented in the electronic health record among the participants at the time of inclusion.

A total of 166 participants were recruited, of whom 120 were aged 40–69 years and were included in the analyses. Seven participants had missing values in one or more variables necessary for the SCORE2 calculation. All participants were seeking care for mental illness. Nine of the included participants did not have any registered diagnosis related to mental illness before the contact was made with the PHCC (S1 Table. Overview of modifiable THD risk factors and psychiatric diagnoses). However, all of the remaining participants had a diagnosis of mental illness prior to inclusion in the study: depression, anxiety, sleeping disorder, burnout, ADHD/ADD/autism, alcohol addiction, or post-traumatic stress syndrome (Fig 1).

Of the 113 patients with complete data aged 40–69, 36% of the women (27/76) and 86% of the men (32/37) had an increased cardiovascular risk (moderate or high) according to SCORE2 (Table 2).

**Table 2:** Measurements by sex and SCORE2 risk group for participants aged 40-69.

Sex (n)	Women (76, missing = 5^1^)				Men (37, missing = 2^1^)			
SCORE2 risk group*	1* (n = 49)	2 **(n = 27)	3*** (n = 0)	P^2^	1* (n = 5)	2** (n = 29)	3*** (n = 3)	P^2^
Age (μ) Median Sd min-max	51.1 50 6.14 40-64	58.9 60 7.0 42-69	– – – –	<0.001	46.2 43 5.9 40-53	52.3 50 7.0 41-64	63 61 4.4 60-68	0.028
BMI (μ) Median Sd min-max	28.7 26.7 7.0 18.6-54.5	29.4 27.6 6.79 19-51.6	– – – –	0.558	25.4 24.7 1.55 24.3-28.1	31.0 30.3 5.28 23.3-44.9	24.8 23.9 3.2 22.1-28.4	0.005
Waist (μ) Median Sd min-max	93.7 90.5 15.9 67-131	94.5 93 14.1 70-129	– – –	.753	91.8 92 7.76 82-102	107.8 108 12.6 83-142	100 98 8.2 93-109	0.020
WHR (μ) Median Sd, min-max	0.86 0.86 0.09 0.70-1.17	0.85 0.85 0.07 0.68-0.99	– – – –	0.943	0.93 0.94 0.05 0.84-0.98	0.98 0.97 0.08 0.83-1.20	1.0 0.97 0.08 0.94-1.09	0.472
Systolic Blood pressure (μ) Median Sd min-max	122.5 123 11.0 100-149	132.5 126 19.8 105-175	– – – –	0.089	116.4 120 10.4 105-130	137.4 136 15.9 110-189	145.7 147 5.1 140-151	0.005
Diastolic Blood pressure (μ) Mmedian Sd min-max	79.6 80 8.7 60-97	84.4 80 11.9 70-110	– – – –	0.186	74.6 80 7.6 64-80	87.1 87 8.6 70-109	88.7 90 2.3 86-90	0.021
Fasting glucose mmol/l (μ) Median Sd min-max	5.4 5.2 0.73 4.1-8.1	5.68 5.4 0.82 4.8-8.4	– – – –	0.135	6.6 (4) 5.65 2.26 5.2-10	6.0 5.65 0.94 4.8-8.3	5.47 5.6 0.32 5.1-5.7	0.639
Cholesterol (μ) Median Sd min-max	4.9 4.9 0.93 3.2-6.8	5.5 5.7 1.09 3.1-7.7	– – – –	0.015	5.0 4.8 0.74 4.4-6.3	4.7 5.0 1.33 2.5-6.7	4.6 4.3 1.39 3.5-6.2	0.918
LDL mmol/l (μ) Median Sd min-max	3.2 3.2 0.99 1.4-5.2	3.5 3.6 0.97 1.6-5.6	– – – –	0.434	3.3 3.2 1.25 1.6-5.1	3.3 3.3 1.27 1-5.7	3.0 3.1 0.75 2.2-3.7	0.901
HDL mmol/l (μ) Median Sd min-max	1.5 1.5 0.33 0.88-2.2	1.8 1.7 0.50 0.97-3	– – – –	0.009	1.29 1.2 0.15 1.1-1.5	1.14 1.0 0.40 .6-2.4	0.8 1.0 0.62 .1-1.3	0.316
Non-HDL mmol/l (μ) Median Sd min-max	3.41 3.3 0.98 1.4-5.1	3.72 3.8 1.03 1.6-6.13	– – – –	0.241	3.74 3.5 0.78 3.2-5.1	3.60 3.5 1.20 1.6-5.6	3.87 4.2 1.23 2.5-4.9	0.914

*SCORE2 risk group 1: age < 50 and <2.5% risk, or age 50–69 and <5% risk.

**SCORE2 risk group 2: age < 50 and 2.5- < 7.5% risk, or age 50–69 and 5- < 10% risk.

***SCORE2 risk group 3: age < 50 and ≥7.5% risk, or age 50–69 and ≥10% risk.

^1^Missing laboratory value to calculate SCORE2

^2^Kruskall-Wallis rank test between SCORE2 levels.

Among women, tobacco use and total cholesterol (both included in the SCORE2 risk assessment) showed a statistically significantly higher distribution in the moderate SCORE2 level compared with the low level (Table 3). Among men, a higher proportion had a BMI > 25 kg/m^2^ among those with moderate SCORE2 risk compared to those with low and high SCORE2 risk (Table 3). A large proportion of patients with a low SCORE2 risk (green) had several other cardiovascular risk factors identified through a THD (including high WHR, LDL > 3.0 mmol/l, and physical inactivity) (Table 3).

**Table 3:** The percentage of participants (40-69 years) exceeding recommended measurement levels in different SCORE2 groups.

Measurements	SCORE2 risk Women (n = 76, missing = 5)				SCORE2 risk Men (n = 37, missing = 2)			
	1* (n = 49)	2** (n = 27)	3*** (n = 0)	P^1^	1* (n = 5)	2 ** (n = 29)	3*** (n = 3)	P^1^
WHR^2^ (%)	54.2	51.9	–	0.848	80.0	79.3	100.0	0.690
BMI > 25 kg/m^2^ (%)	66.7	81.5	–	0.173	40.0	93.1	33.3	0.002
Heredity CVD^3^ (%)	25.5	18.5	–	0.493	20.0	20.7	0.0	0.689
Heredity DM^3^ (%)	37.5	30.4	–	0.574	33.3	40.7	0.0	0.389
Total Cholesterol >5 mmol/l (%)	46.8	73.1	–	0.032	20.0	50.0	33.3	0.540
HDL^4^ (%)	26.5	11.1	–	0.117	0.0	34.5	33.3	0.304
LDL > 3.0 mmol/l (%)	53.1	66.7	–	0.253	60.0	62.1	66.7	0.983
Non-HDL > 3.8 mmol/l (%)	34.7	44.4	–	0.405	20.0	41.4	66.7	0.430
Alcohol^5^ (%)	10.2	22.2	–	0.157	20.0	17.2	66.7	0.148
Nicotine use Snus and e-cigs (%)	2.1	4.6	–	0.580	40.0	19.2	0.0	0.448
Smoking tobacco (%)	4.2	19.2	–	0.036	0.0	12.5	33.3	0.481
Any nicotine use (%)	6.1	22.2	–	0.039	40.0	27.6	33.3	0.850
Unhealthy diet^6^ (%)	45.8	63.0	–	0.221	0.0	48.3	66.7	0.180
Physical activity <150 min/week (%)	62.2	65.0	–	0.861	33.3	70.0	100.0	0.437
Systolic blood pressure >130 mmHg (%)	26.1	40.0	–	0.229	0.0	69.2	100.0	0.012
Diastolic blood pressure >80 mmHg (%)	51.3	61.9	–	0.434	0.0	80.8	100.0	0.022

*SCORE2 risk group 1: age < 50 and <2.5% risk, or age 50–69 and <5% risk.

**SCORE2 risk group 2: age < 50 and 2.5- < 7.5% risk, or age 50–69 and 5- < 10% risk.

***SCORE2 risk group 3: age < 50 and ≥7.5% risk, or age 50–69 and ≥10% risk.

^1^Kruskall-Wallis rank test between SCORE2 levels.

^2^WHR ≤ .85 for women and ≤.90 for men.

^3^Heredity = at least one first-grade relative with diabetes mellitus type 2. Cardiovascular disease among male first-degree relatives before age 70, and women first-degree relatives before age 75.

^4^HDL < 1.0 for men and <1.3 for women.

^5^Alcohol >10 glasses of 4 cl alcohol/ week or >4 glasses of 4 cl alcohol on the same occasion.

^6^According to Nordic Nutrition Recommendations 2023 [22]

## Discussion

### Main findings

Our study showed that in this population with psychiatric diagnoses in primary care, 36% of the women and 86% of the men had a moderate or high cardiovascular risk according to SCORE2. Many patients with an increased SCORE2 risk also had several other elevated cardiovascular risk factors identified through the THD, such as BMI > 25 kg/m^2^, elevated WHR and an unhealthy diet. More than half of the women in the low SCORE2 risk group had high WHR, BMI, LDL > 3 mmol/l, were physically inactive, and had elevated diastolic blood pressure. Based on these results, THD seems to add value to the risk assessment with SCORE2 in this population.

### Comparison with other studies

In contrast to previous research, our study is the first to assess SCORE2 in a primary care mental‑health population and to compare SCORE2 with THD as complementary risk assessment tools.

Several regions in Sweden use the THD model to promote healthy lifestyle habits and reduce the risk of incident disease by screening specific age groups for unhealthy lifestyle habits. Meanwhile, previous research has shown that certain groups, for example, people with mental illness, have a higher prevalence of unhealthy lifestyle habits compared with the general age-matched population [10–12]. Additional recent large-scale evidence supports this pattern; a prospective cohort study from the All of Us Research Program (n ≈ 120,000) reported that depression, anxiety, bipolar disorder and PTSD all independently increased the risk of incident cardiovascular disease, especially heart failure, even after adjustment for SCORE2-relevant factors, with effects particularly pronounced among women [23]. Furthermore, a 2025 analysis in The Lancet Regional Health – Europe highlighted that psychosocial stress, inflammation, and healthcare disparities substantially contribute to the excess cardiovascular risk observed in individuals with mental health disorders, reinforcing the limitations of traditional risk prediction models in these groups [24].

A recent Swedish study on THD in 40-year-old individuals showed that, according to SCORE2, 25% of the men were at moderate risk of developing a cardiovascular event within 5–10 years, but only 2% of the women. Our results showed a higher proportion of individuals having moderate or high cardiovascular disease (CVD) risk. This may be attributed not only to the expected higher CVD risk associated with mental illness, but also to the higher average age within this group. In Sweden, approximately 50% of men and 80% of women aged 40–69 years have a low 10-year risk according to SCORE2 [3]. In our study, a lower proportion of individuals had a low estimated 10-year risk according to the recalibrated SCORE2: ~ 14% of the men and ~65% of the women. Our study’s high mean age (50 years) might partly explain the difference. However, a recent study also showed that SCORE2 might underestimate the 10-year CVD risk among patients with depression and anxiety [25], and that this could be attributable to their distinct characteristics that SCORE2 could not capture. It is also stated by the 2025 ESC Clinical Consensus Statement on mental health and cardiovascular disease explicitly recommends integrating mental health assessment into cardiovascular risk evaluation and identifies mental illness as a risk-enhancing factor [26]. Consistent with these recommendations, a 2024 European Heart Journal analysis on severe mental illness demonstrated that conventional cardiovascular risk prediction models underestimate long-term cardiovascular risk in patients with severe mental illness, underscoring the need for complementary or adapted assessment tools in this population [27]. In this context, a complementary case, other risk assessment instruments, such as THDs, may be better suited to identify additional modifiable lifestyle-related risk factors not included in SCORE2 [23,28].

There is ongoing debate about whether healthcare should intervene to promote lifestyle changes and if the recommendations are harmful or sustainable over time [29,30]. Because many individuals have unhealthy lifestyle habits, broad screening programs or interventions can be resource-demanding in a strained primary care setting [31]. Based on the existing evidence [9,32] and our present and previous results [10,11], we argue that individuals with psychiatric illness in primary care should be prioritised for such interventions. Even if THD is more time-consuming than a quick risk assessment with SCORE2, we have demonstrated in this study that THD can identify several cardiovascular risk factors that are not included in the SCORE2 risk assessment. Unlike SCORE2, THD includes motivational dialogue to promote a healthy lifestyle. Motivational interviewing has been shown to be an effective tool for helping individuals change behaviours [33,34], and can be used regardless of age for preventive purposes. When the participants with mental illness, in a pilot and feasibility study using THD, were followed up after approximately a year, most showed improvements in at least one lifestyle habit [11]. Other studies have also shown that health-related quality of life and physical activity increase after a THD [35].

### Strengths and limitations

A key strength of this study is that it is, to our knowledge, the first to assess cardiovascular risk using SCORE2 in a population with mental illness in primary care. But also, the first study that compares SCORE2 with THD in this context. One major strength of the study is the opportunistic method of inclusion, as it reflects real-world primary care. Despite higher cardiovascular risk, patients with mental illness could be challenging to reach through common health screening programs. In our study, all caregivers (physicians, nurses, psychologists) who had contact with the patient could recruit them, minimising recruitment bias. However, our results can only be applied to individuals seeking care due to mental illness, and not to those who cope with their symptoms by themselves. The study’s main limitation is the small sample of included patients, especially men. Despite the small sample size of this study, it supports the hypothesis that this patient group requires closer monitoring of lifestyle factors that may impact their well-being and increase the risk of developing diabetes or cardiovascular disease. Another limitation is the lack of data on the participants’ socioeconomic status. Socioeconomic status is linked to higher rates of tobacco use, physical inactivity, and poor nutrition [36]. Because the design was cross-sectional, no longitudinal outcomes were captured, and no information was collected on behavioural change or clinical follow-up after the THD. Therefore, it is not possible to determine whether the identified lifestyle risk factors were subsequently addressed or modified.

SCORE2 is validated only for individuals aged 40–69 years; cardiovascular disease and mental illness burden extend beyond this age range, which limits generalisability. Furthermore, THD does not assess certain lifestyle risks such as recreational or illicit drug use. This represents an unmeasured confounder. Finally, the study was conducted as an exploratory pilot. Given the limited sample size, multivariable regression modelling was not appropriate; therefore, only descriptive and non-parametric analyses were performed.

Nonetheless, the patients were recruited from six different primary health care centres in rural and urban areas, with varying socioeconomic statuses. This indicates that the results could be generalised to a broad socioeconomic context. However, our findings are limited to patients seeking primary care for mental illness and cannot be generalised to patients with severe mental illnesses.

## Conclusion

In conclusion, Targeted Health Dialogues may complement SCORE2 by identifying additional modifiable lifestyle-related risk factors relevant to cardiovascular prevention in patients with mental illness. Further research, including longitudinal and interventional designs, is warranted to evaluate whether THD-guided lifestyle counselling can reduce cardiovascular risk in this population.

## Supporting information

S1 Table Overview of lifestyle-related cardiovascular risk factors assessed in the Targeted Health Dialogue (THD) and psychiatric diagnoses among study participants. This table includes all modifiable lifestyle factors evaluated in the THD (e.g., BMI, WHR, physical activity, diet quality, alcohol use, nicotine use, sleep, fasting glucose, lipids, blood pressure, family history) and all psychiatric diagnoses represented in the cohort (depression, anxiety disorders, stress-related disorders, insomnia, ADHD/ADD/autism spectrum disorders, alcohol use disorder, PTSD).
(DOCX)

## Abbreviations

SCORE2: Systematic COronary Risk Evaluation 2
THD: Targeted health dialogue;
CVD: Cardiovascular disease
DM2: Diabetes mellitus type 2
WHR: Waist hip ratio
BMI: Body mass index
LDL: Low-density lipoprotein
HDL: High-density lipoprotein
