# Supplementary material for: Cardiovascular risk assessment with SCORE2 and targeted health dialogues in primary care patients with mental illness
Source: PLoS One. 2026 May 4;21(5):e0348365. doi: 10.1371/journal.pone.0348365 (PMC13138616; doi:10.1371/journal.pone.0348365)
Supplement: S1 Table — This table includes all modifiable lifestyle factors evaluated in the THD (e.g., BMI, WHR, physical activity, diet quality, alcohol use, nicotine use, sleep, fasting glucose, lipids, blood pressure, family history) and all psychiatric diagnoses represented in the cohort (depression, anxiety disorders, stress-related disorders, insomnia, ADHD/ADD/autism spectrum disorders, alcohol use disorder, PTSD). (DOCX) [file pone.0348365.s001.docx]

Table S.1 Overview of modifiable THD risk factors and psychiatric diagnoses

A. Modifiable lifestyle-related cardiovascular risk factors assessed in THD:

| Category | THD Variable |
| --- | --- |
| Anthropometrics | BMI, Waist–Hip Ratio |
| Vital signs | Systolic BP, Diastolic BP |
| Laboratory values | Fasting glucose, Total cholesterol, LDL, HDL, Non‑HDL |
| Lifestyle behaviours (questionnaires) | Diet quality, Physical activity, Smoking, Snus, Vaping, Alcohol use, Sleep quality |
| Family history | CVD, Diabetes mellitus type 2 |

B. Psychiatric diagnoses represented in the study population:

| Diagnostic group | Diagnosis included |
| --- | --- |
| Affective/anxiety | Depression, Generalised anxiety disorder |
| Stress-related | Stress, Exhaustion, Burnout |
| Sleep disorders | Insomnia |
| Neurodevelopmental | ADHD, ADD, Autism spectrum disorders |
| Trauma-related | PTSD |
